# Comparative evaluation of root canal obturation techniques and sealers in Diabetic Dentin: An in-vitro push-out bond strength test

**DOI:** 10.1371/journal.pone.0353975

**Published:** 2026-07-17

**Authors:** Mevlüt Sinan Ocak, Mehmet Eskibağlar, Mustafa Gündoğar

**Affiliations:** 1 Firat University, Faculty of Dentistry, Department of Endodontics, Elazig, Turkey; 2 Department of Endodontics, Faculty of Dentistry, Istanbul Medipol University, Istanbul, Turkey; University of Puthisastra, CAMBODIA

## Abstract

This study evaluated the comparative performance of different root canal obturation approaches using different sealing materials on the push-out bond strength of dentin samples obtained from patients with diabetes mellitus (DM). Forty single-rooted mandibular premolars were instrumented using the Reciproc R40 system and randomly assigned to four groups (n = 10 teeth each): (1) cold lateral compaction with AH Plus, (2) warm vertical compaction with AH Plus, (3) hydraulic single-cone obturation with gutta-percha and Bioserra sealer, and (4) sealer-only technique using the Bioserra bioceramic sealer. After storage at 37°C and 100% humidity for one week, 1-mm slices were prepared from the coronal, middle, and apical thirds and subjected to push-out testing. Data were analyzed using ANOVA and Tukey’s test (p < 0.05). Significant differences in push-out bond strength were observed among the obturation approaches using different sealer materials (p < 0.05). Bioceramic sealers exhibited higher bond strengths than epoxy resin–based sealers, particularly in the middle and apical thirds. Hydraulic single-cone and bioceramic sealer-only approaches outperformed conventional techniques (p < 0.001), whereas no significant difference was observed between cold lateral and warm vertical compaction techniques. Obturation approaches employing bioceramic sealers demonstrated superior bonding in diabetic dentin, suggesting potential advantages over conventional methods in the context of diabetic dentin.

## Introduction

Diabetes mellitus (DM) is a complex multisystemic metabolic disorder projected to affect nearly 853 million people by 2050. Currently, more than 589 million adults aged 20–79 years live with DM worldwide, reflecting a significant global health burden [1]. DM is characterized by persistent hyperglycemia and arises from a combination of genetic and environmental factors [2]. It exerts systemic effects on multiple organs and tissues, including the oral cavity [3]. Previous studies have shown that poor glycemic control can negatively affect the dental pulp, connective tissues, and cellular structures, thereby compromising oral health [4–6]. Dentin, a mineralized tissue composed of approximately 70% inorganic material, 20% organic matrix, and 10% water, is highly relevant for endodontic treatment [7]. DM alters the trace element composition of dentin, particularly strontium, magnesium, zinc, and selenium, within the hydroxyapatite structure. These changes may affect the crystallinity and physicochemical characteristics of the mineral phase, potentially weakening the biomechanical properties of dentin, reducing microhardness, lowering fracture resistance, and impairing adhesion capacity of root canal filling materials [8,9].

Given these substrate alterations, the interaction between dentin and obturation materials may depend on the material used. Calcium silicate–based bioceramic sealers release calcium ions and promote biomineralization at the sealer–dentin interface, resulting in the formation of hydroxyapatite-like interfacial structures and chemical interactions with the inorganic phase of dentin [10–12]. Therefore, in mineral-altered diabetic dentin, it may be hypothesized that bioceramic sealers may establish enhanced substrate interactions through ionic exchange and interfacial mineral deposition compared with epoxy resin–based sealers, which rely predominantly on micromechanical retention and limited chemical bonding [12]. This potential difference in the bonding mechanism may be particularly relevant in structurally compromised dentin affected by chronic hyperglycemia [9,13].

Root canal obturation aims to achieve a fluid-tight seal along the root canal system, commonly using gutta-percha (GP) in combination with a sealer. Traditional techniques, such as cold lateral compaction, are still widely used but have limitations, including insufficient adaptation to the canal walls and potential root fractures due to microcrack formation. Warm vertical compaction improves adaptation but remains technique sensitive [14, 15]. In contrast, newly developed calcium silicate–based bioceramic sealers, such as Bioserra, which is a premixed sealer containing tricalcium silicate and dicalcium silicate as bioactive components, together with calcium aluminate, calcium aluminum oxide, tricalcium aluminate, and tantalum as radiopacifiers, have demonstrated superior push-out bond strength compared to resin-based sealers in healthy dentin [16]. However, their performance in structurally compromised diabetic dentin remains unclear.

One of the main objectives of root canal obturation is to reinforce the root dentin and increase its resistance to fracture [17]. It has been suggested that materials that adhere to the dentin surface can contribute to strengthening the remaining tooth structure [18]. The push-out bond strength test is widely used to evaluate the adhesion between obturation materials and canal walls because of its simplicity and reproducibility [19]. This test reflects a combination of frictional forces, molecular interactions, and chemical bonding between the materials and dentinal walls [20].

A review of the available literature indicates that, although several studies have assessed the bonding ability of individual materials, such as mineral trioxide aggregate (MTA) or specific sealers, in diabetic dentin, [13,21]. limited evidence exists regarding the influence of different obturation techniques on bond strength in this altered substrate. Most previous investigations have focused primarily on material properties rather than technique-dependent variables. Furthermore, the current literature highlights the limited and heterogeneous nature of data concerning the mechanical performance of diabetic dentin [9]. To our knowledge, no study has directly compared multiple root canal obturation approaches in diabetic dentin under standardized conditions while isolating the intrinsic bonding potential of sealers. Therefore, this study aimed to evaluate the influence of various obturation techniques and sealer types on the push-out bond strength in dentin derived from patients with diabetes. This study did not compare diabetic and healthy dentin. Instead, it focused exclusively on dentin derived patients with diabetes to evaluate the relative performance of different obturation techniques under clinically compromised conditions, allowing the assessment of intragroup differences without additional confounding factors.

Therefore, this study aimed to compare the push-out bond strengths of different root canal obturation approaches using different sealing materials in teeth obtained from patients with diabetes mellitus. A sealer-only group was included to isolate and assess the direct interaction between the sealer and dentin, independent of the influence of gutta-percha. The null hypothesis (H₀) was that there would be no significant difference in the push-out bond strength among the tested obturation techniques.

## Materials and methods

### Sample selection

The required sample size was calculated using GPower software (GPower 3.1.7, Windows, Düsseldorf, Germany) to obtain eight specimens per group. To increase the robustness of the analysis and compensate for potential specimen loss, the final sample size was increased to 10 specimens per group (40 teeth).

After obtaining ethical approval from the Firat University Non-Interventional Ethics Committee (Decision no: 2023/10–31), single-rooted mandibular premolar teeth extracted from patients diagnosed with diabetes mellitus (DM) and each possessing a single round canal were collected and examined visually and radiographically. All teeth were extracted for clinical reasons unrelated to the objectives of this study, such as periodontal and orthodontic indications. Written informed consent was obtained from all patients permitting the use of their extracted teeth for research purposes. All procedures were performed in accordance with the ethical principles outlined in the World Medical Association Declaration of Helsinki for medical research involving human tissue specimens, with strict protection of donor anonymity and confidentiality. The recruitment period for this study started on 31/10/2023 and ended on 31/10/2024. Teeth with prior root canal treatment, cracks, or caries were excluded. Soft tissue and debris were removed from the collected teeth using a curette. Owing to limitations in patient records, no data were available regarding the type of diabetes or the disease duration. Only patients with documented diabetes mellitus and HbA1c levels ≥ 6.5% obtained from their medical records at the time of tooth extraction were included in the study [1].

To standardize the root lengths, the roots were sectioned under water cooling with a diamond disc to obtain a 12 millimeters (mm) segment from the apex. Canal patency was confirmed using a #15 K-file (Dentsply/Maillefer, Ballaigues, Switzerland). The root canals were then instrumented using the Reciproc system (VDW, Munich, Germany) to size R40. During the shaping procedure, 10 mL of 2.5% sodium hypochlorite (NaOCl) was used per canal, delivered incrementally using a flexible, side-vented irrigation needle. After instrumentation, the smear layer was removed using 1 mL of 17% ethylenediaminetetraacetic acid (EDTA) for 1 min. Final irrigation was performed using 2 mL of distilled water, and the canals were dried using sterile paper points.

### Root canal obturation

Randomization was performed at the tooth level using a simple random allocation method prior to sectioning. Ten teeth were allocated to each experimental group. Each tooth was assigned to a single group to ensure a balanced distribution and prevent intra-tooth correlation across groups. All obturation procedures were performed by a single experienced operator using various techniques.

- In Group 1, cold lateral compaction was performed using an AH Plus sealer (Dentsply DeTrey GmbH, Konstanz, Germany).
- In Group 2, warm vertical compaction was performed using the AH Plus sealer and the Elements Free cordless obturation system (SybronEndo/Kerr Endodontics, Orange, CA, USA).
- In Group 3, single-cone obturation was performed using gutta-percha and Bioserra sealer (Meta Biomed, Dongsaeng Myeong 1-ro, Korea).
- In Group 4, the root canals were filled exclusively with the Bioserra sealer without the use of gutta-percha to evaluate the intrinsic bonding ability of the sealer to root dentin.

To ensure complete setting of the root canal sealers, the specimens were stored at 37°C and 100% humidity for one week. After obturation, standardized slices of 1 mm thickness were obtained from the coronal, middle, and apical thirds (at 2.5, 5, and 8 mm from the apex, respectively) using a water-cooled diamond saw (Isomet, Buehler Ltd., USA).

### Push-out bond strength test

**Fig 1:**
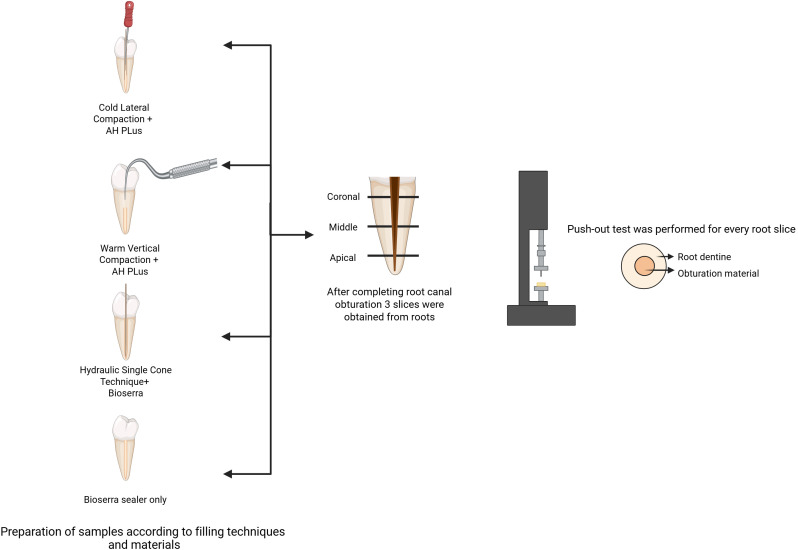
Sample preparation and push-out testing of root slices after obturation using different techniques and materials.

The 120 slices were subjected to a push-out test using a universal testing machine (Instron Industrial Products, Norwood, MA, USA) at a crosshead speed of 1 mm/min in the apicocoronal direction (Fig 1). Different plungers were used for the coronal, middle, and apical sections to ensure a proper fit within the canal space and avoid contact with the surrounding dentin. Each specimen was positioned with its apical side facing the plunger to minimize the stress on the dentin.

The force required to dislodge the filling material (in kilonewtons (kN)) was converted to stress (in megapascals (MPa)) [22]. The upper and lower diameters of each specimen were measured individually using the following formula:


$$\begin{array}{c} MPa=F/SL, \end{array}$$

where SL = sealer adhesion area = π(R + r)g,

π = 3.14, R = coronal canal radius (mm), r = apical canal radius (mm), and g = slice thickness (mm).

Dislodgement of the filling was confirmed by a sudden drop in the load–displacement curve. The push-out bond strength was calculated for each root slice. For statistical analysis, the mean push-out bond strength values obtained from the coronal, middle, and apical slices of each tooth were calculated.

### Data analysis

Statistical analyses were performed using IBM SPSS Statistics for Windows, Version 23.0 (SPSS Inc., Chicago, IL, USA). For statistical analysis, the mean push-out bond strength value per tooth was considered as the statistical unit. The normality of the data was assessed using the Kolmogorov–Smirnov and Shapiro–Wilk tests, and the homogeneity of variances was verified using Levene’s test. For normally distributed data with equal variances, one-way ANOVA was used to compare the push-out bond strength values, followed by Tukey’s HSD post hoc test for pairwise comparisons. The results are presented as mean ± standard deviation, with a significance level set at p < 0.05.

## Results

No statistically significant differences were observed between the Cold Lateral and Warm Vertical groups in the coronal, middle, or apical thirds (p > 0.05; Table 1). In contrast, both the hydraulic single cone and bioceramic sealer-only groups exhibited significantly higher push-out bond strength values in all root regions than the conventional techniques (p < 0.001; Table 1). A significant difference was also detected between the hydraulic single cone

**Table 1:** Push-out bond strength (MPa) according to obturation technique and root region (mean ± SD).

Coronal					Middle					Apical				
	N	Mean	Std. Deviation	P*		N	Mean	Std. Deviation	P*		N	Mean	Std. Deviation	P*
Cold Lateral	10	3.716	0.678	<0.001	Cold Lateral	10	4.731	0.545	<0.001	Cold Lateral	10	4.469	0.746	<0.001
Warm Vertical	10	3.249	0.701		Warm Vertical	10	5.742	0.889		Warm Vertical	10	5.444	1.016	
Hydraulic ^a1,b1^	10	5.086	0.655		Hydraulic ^a1,b1^	10	9.246	0.938		Hydraulic ^a1,b1^	10	8.787	1.097	
Bioceramic ^a2,b2,c^	10	9.140	0.985		Bioceramic ^a,b,c^	10	13.123	1.408		Bioceramic ^a,b,c^	10	11.101	1.332	

* All groups were analyzed using one-way ANOVA, followed by Tukey’s HSD post hoc test. For each root region (coronal, middle, and apical), different lowercase letters (a, b, c) indicate statistically significant differences between the obturation techniques (p < 0.05). Numerical superscripts (1, 2) indicate statistically significant differences among the root regions within the same obturation technique (p < 0.05).

and bioceramic sealer-only groups in the coronal region, with higher values observed in the bioceramic sealer-only group (p < 0.05; Table 1).

In the intragroup analysis of the bioceramic sealer-only group, bond strength values in the middle and apical thirds were significantly higher than those in the coronal third (p < 0.001). Moreover, a significant difference was observed between the middle and apical thirds (p < 0.001; Table 2). A similar pattern was observed in the hydraulic single cone group, with significantly higher values in the middle and apical thirds than in the coronal third (p < 0.001). This trend was also observed in the Warm Vertical and Cold Lateral groups, although with a lower magnitude and statistical significance (p < 0.05; Table 2).

**Table 2:** Regional comparison of push-out bond strength (MPa) within each obturation technique (mean ± SD).

Cold Lateral					Warm Vertical					Hydraulic					Bioceramic				
	N	Mean	Std. Deviation	P*		N	Mean	Std. Deviation	P*		N	Mean	Std. Deviation	P*		N	Mean	Std. Deviation	P*
Coronal	10	3.716	0.678	<0.001	Coronal	10	3.249	0.701	<0.001	Coronal	10	5.086	0.655	<0.001	Coronal	10	9.140	0.985	<0.001
Middle ^a1^	10	4.731	0.545		Middle ^a1^	10	5.742	0.889		Middle ^a1^	10	9.246	0.938		Middle ^a1^	10	13.123	1.408	
Apical ^a2^	10	4.469	0.746		Apical ^a2^	10	5.444	1.016		Apical ^a2^	10	8.787	1.097		Apical ^a2,b^	10	11.101	1.332	

*All groups were analyzed using one-way ANOVA, followed by Tukey’s HSD post hoc test. Within each obturation technique, different lowercase letters indicate statistically significant differences among the root regions. Numerical superscripts indicate statistically significant differences among the obturation techniques within the same root region (p < 0.05).

When the obturation techniques were compared across all root regions, both the bioceramic sealer-only and hydraulic single cone groups showed significantly higher overall push-out bond strengths than the conventional groups (p < 0.001; Table 3). Bioceramic sealer-only group demonstrated the highest overall mean bond strength, followed in descending order by the Hydraulic Single Cone, Warm Vertical, and Cold Lateral groups.

**Table 3:** Overall comparison of push-out bond strength (MPa) among obturation techniques (mean ± SD).

	N	Mean	Std. Deviation	P*
Cold Lateral	10	4.305	0.774	<0.001
Warm Vertical	10	4.811	1.413	
Hydraulic ^a,b^	10	7.706	2.090	
Bioceramic ^a,b,c^	10	11.121	2.050	

*Statistical analysis was performed using one-way ANOVA, followed by Tukey’s HSD post hoc test. Different lowercase letters (a, b, c) indicate statistically significant differences between obturation techniques (p < 0.05).

## Discussion

This study investigated the influence of various root canal obturation techniques and sealer types on the push-out bond strength in dentin derived from patients with diabetes. Chronic hyperglycemia in diabetes promotes the accumulation of advanced glycation end-products (AGEs), which can disrupt collagen integrity [9]. Diabetes has been associated with an increased susceptibility to vertical root fractures [23], which may indirectly reflect alterations in dentin mechanical behavior. These changes are generally associated with impaired adhesion of resin-based materials, as hybrid layer formation depends on collagen integrity [24,25]. Therefore, AGE-induced alterations in dentin collagen could potentially influence the bonding performance of epoxy resin–based sealers [21, 26]. In contrast, calcium silicate–based bioceramic sealers rely less on collagen-dependent micromechanical retention and more on ionic exchange and biomineralization with the inorganic phase of dentin [10–12]. This difference in the bonding mechanism may explain the superior performance of the bioceramic materials observed in the present study. However, limited data exists regarding how such alterations affect the adhesion of root canal sealers in diabetic dentin [13,27]. Although previous studies have demonstrated the bonding potential of bioceramic sealers to healthy dentin through chemical interactions [11,28], the present study uniquely evaluated their performance in biochemically compromised dentin under clinically relevant conditions.

The results demonstrated that both the obturation technique and sealer type significantly impacted the push-out bond strength. Groups utilizing bioceramic sealers exhibited consistently higher bond strength across all root sections than those utilizing techniques (p < 0.001), suggesting that the chemical bonding capacity and bioactivity of calcium silicate–based materials may offer distinct advantages in diabetic dentin.^10^

In the coronal region, the bioceramic sealer-only group demonstrated a significantly higher bond strength than the hydraulic single cone group (p < 0.001; Table 1). This difference may be attributed to the more homogeneous sealer mass and greater interface adaptation achieved with the bioceramic technique, which enhances chemical bonding and reduces the formation of voids. In support of this, previous studies have shown that calcium silicate–based sealers exhibit superior dentinal tubule penetration and promote biomineralization owing to their hydrophilic and bioactive properties, which reinforce interfacial bonding over time [10,11,29].

Within the Bioceramic sealer-only group, the bond strength values in the middle and apical thirds were significantly higher than those in the coronal region (p < 0.001; Table 2), which is consistent with previous studies reporting increased push-out bond strength toward the middle and apical root thirds for calcium silicate–based sealers [30,31]. This finding may be related to regional differences in dentin morphology and canal geometry. Although apical dentin generally presents with narrower dentinal tubules, the reduced canal diameter and increased confinement of the filling material may enhance frictional resistance and sealer adaptation in the apical sections [12]. Additionally, diabetes-induced alterations in the dentin structure, including changes in collagen integrity and mineral composition associated with AGEs, may influence bonding behavior in a region-dependent manner [13]. However, these proposed explanations remain hypothetical, as the present study did not directly assess the regional structural or biochemical differences in diabetic dentin.

In the hydraulic single cone group, bond strength values in the middle and apical thirds were significantly higher than those in the coronal third (p < 0.05; Table 2). Conversely, Cold Lateral and Warm Vertical compaction showed significantly lower bond strengths than bioceramic-based approaches (p < 0.05; Table 3). These findings indicate that resin-based sealers may offer limited adhesion to structurally compromised diabetic dentin, likely because they rely on mechanical interlocking rather than chemical bonding [32].

Bioceramic sealers demonstrated the highest bond strength values in the middle and apical thirds, further confirming their superior adhesion characteristics in root regions with narrower tubules and higher surface areas (p < 0.001; Table 2). These results highlight the potential clinical relevance of calcium silicate–based sealers in diabetic dentin, where microstructural and biochemical changes may compromise the effectiveness of conventional obturation systems.

Although the standard deviation values did not exceed the mean values, a relatively greater dispersion was observed in certain groups, particularly those involving bioceramic sealers. This variability may reflect the heterogeneity of dentin substrate quality among individuals with diabetes [9,13]. Chronic hyperglycemia does not affect dentin uniformly; variations in disease duration and metabolic history may lead to heterogeneous structural and biochemical alterations [9]. Such changes can influence the mineral composition, collagen integrity, and biomechanical behavior of dentin [13], which may partially account for the variability observed in the push-out bond strength measurements in this study.

This study had several limitations that should be considered when interpreting the findings. First, the specimens were collected from diabetic patients of variable ages and disease durations, and age and gender were not included as controlled variables within the scope of this experimental design, which may have contributed to heterogeneity in dentin structure and mechanical behavior [9,13]. The influence of age and gender on the results was not evaluated. Second, the push-out bond strength test was conducted in vitro, lacking physiological conditions such as masticatory loading, thermal cycling, and salivary interactions, which may affect long-term performance [33]. Third, only single-rooted canals with a standardized anatomy were used, and variations in canal morphology or irrigation protocols were not explored. Another limitation is that obturation techniques were evaluated in combination with different sealer materials, which does not allow complete isolation of the individual effect of each variable. However, this approach was intentionally adopted to reflect clinically relevant treatment strategies, where specific techniques are commonly used with particular types of sealers. Finally, although previous studies have included non-diabetic control groups to characterize diabetes-related dentin changes [13,27], the present study focused exclusively on diabetic samples to isolate the effects of the obturation techniques. These limitations warrant caution when generalizing the results to broader clinical scenarios.

Despite these limitations, the present study provides valuable insights into the bonding behavior of root canal obturation materials in a clinically relevant diabetic dentin model, addressing a significant gap in endodontic literature. Future studies should explore the biochemical mechanisms underlying sealer–dentin interactions in diabetes, including the role of advanced glycation end-products and collagen alterations. The absence of a non-diabetic control group should be considered when interpreting the results, as the primary objective of the present study was not to compare diabetic and non-diabetic dentin but to evaluate the relative performance of different obturation techniques in a diabetic population. Aging protocols that simulate intraoral conditions (e.g., thermocycling and mechanical loading) are also required to assess long-term material performance. Although diabetes was confirmed using HbA1c values of ≥ 6.5%, detailed information regarding diabetes type, disease duration, and long-term metabolic control was not available, which should be considered when interpreting these results.

From a clinical standpoint, the interaction between dentin substrate quality and sealer characteristics may be particularly relevant in patients with systemic conditions such as diabetes. Although the present findings are based on in vitro conditions, the improved bonding performance of bioceramic sealers in diabetic dentin suggests that material selection may play a critical role in endodontic treatment outcomes in medically compromised patients. The findings are specific to diabetic dentin and should not be extrapolated to healthy substrates; however, a comparative evaluation of obturation techniques within compromised dentin remains clinically relevant.

## Conclusions

Within the limitations of this in vitro study, both the obturation technique and sealer type significantly influenced bond strength in diabetic dentin. Bioceramic-based obturation exhibited superior adhesion compared to conventional methods, underscoring its potential clinical relevance in the endodontic treatment of diabetic dentin.

## Supporting information

S1 Data Raw push-out bond strength values (MPa) for all specimens. The dataset includes individual measurements for each tooth and root region (coronal, middle, apical), along with group allocation. These values were used to calculate the mean and standard deviation reported in the manuscript and to perform the statistical analyses.
(XLSX)
