# Supplementary material for: Comparative evaluation of root canal obturation techniques and sealers in Diabetic Dentin: An in-vitro push-out bond strength test
Source: PLoS One. 2026 Jul 17;21(7):e0353975. doi: 10.1371/journal.pone.0353975 (PMC13378988; doi:10.1371/journal.pone.0353975)
Supplement: Attachment — Submitted filename: PONE-D-26-12466 (1) Jyoti.pdf [file pone.0353975.s002.pdf]

PLOS One

Comparative Evaluation of Root Canal Obturation Techniques and Sealers in Diabetic Dentin: An in-vitro Push-Out Bond Strength Test

--Manuscript Draft--

**Manuscript Number:**	PONE-D-26-12466
**Article Type:**	Research Article
**Full Title:**	Comparative Evaluation of Root Canal Obturation Techniques and Sealers in Diabetic Dentin: An in-vitro Push-Out Bond Strength Test
**Short Title:**	Obturation Methods in Diabetic Root Dentin
**Corresponding Author:**	Mevlüt Sinan OCAK<br>Fırat Üniversitesi: Firat Universitesi<br>Elazığ, TÜRKIYE
**Keywords:**	Bioceramic Sealer; Dentin Adhesion; Diabetes mellitus; Root Canal Obturation; Push-Out Bond Strength
**Abstract:**	This study evaluated the comparative performance of different root canal obturation approaches using different sealer materials on the push-out bond strength of dentin samples obtained from patients with diabetes mellitus (DM). A total of 40 single-rooted mandibular premolars were instrumented using the Reciproc R40 system and randomly assigned to four groups (n=10 teeth each): (1) cold lateral compaction with AH Plus, (2) warm vertical compaction with AH Plus, (3) hydraulic single-cone obturation with gutta-percha and Bioserra sealer, and (4) sealer-only technique using the Bioserra bioceramic sealer. After storage at 37°C and 100% humidity for one week, 1-mm slices were prepared from the coronal, middle, and apical thirds and subjected to push-out testing. Data were analyzed using ANOVA and Tukey's test ($p < 0.05$). Significant differences in push-out bond strength were observed among the obturation approaches employing different sealer materials ($p < 0.05$). Bioceramic sealers exhibited higher bond strengths than epoxy resin–based sealers, particularly in the middle and apical thirds. Hydraulic single-cone and bioceramic sealer-only approaches outperformed conventional techniques ($p < 0.001$), whereas no significant difference was observed between cold lateral and warm vertical compaction. Obturation approaches employing bioceramic sealers demonstrated superior bonding in diabetic dentin, suggesting potential advantages over conventional methods within the context of diabetic dentin.
**Order of Authors:**	Mevlüt Sinan OCAK
	Mehmet ESKİBAĞLAR
	Mustafa GÜNDOĞAR
**Additional Information:**	
**Question**	**Response**
**Financial Disclosure**<br><br>Enter a financial disclosure statement that describes the sources of funding for the work included in this submission. Review the submission guidelines for detailed requirements. View published research articles from *PLOS ONE* for specific examples.<br><br>This statement is required for submission and **will appear in the published article** if	The author(s) received no specific funding for this work.

the submission is accepted. Please make sure it is accepted.<br><br>**Funded studies**<br>Enter a statement with the following details:<br>• Initials of the authors who received each award<br>• Grant numbers awarded to each author<br>• The full name of each funder<br>• URL of each funder website<br>• Did the sponsors or funders play any role in the study design, data collection and analysis, decision to publish, or preparation of the manuscript?<br><br>Did you receive funding for this work?	
**Competing Interests**<br><br>Use the instructions below to enter a competing interest statement for this submission. On behalf of all authors, disclose any competing interests that could be perceived to bias this work—acknowledging all financial support and any other relevant financial or non-financial competing interests.<br><br>This statement is required for submission and **will appear in the published article** if the submission is accepted. Please make sure it is accurate and that any funding sources listed in your Funding Information later in the submission form are also declared in your Financial Disclosure statement.<br><br>View published research articles from *PLOS ONE* for specific examples.	The authors have declared that no competing interests exist.

**NO authors have competing interests**<br><br>Enter: *The authors have declared that no competing interests exist.*<br><br>**Authors with competing interests**<br><br>Enter competing interest details beginning with this statement:<br><br>*I have read the journal's policy and the authors of this manuscript have the following competing interests: [insert competing interests here]*<br><br>* typeset	
**Ethics Statement**<br><br>Enter an ethics statement for this submission. This statement is required if the study involved:<br><br>• Human participants<br>• Human specimens or tissue<br>• Vertebrate animals or cephalopods<br>• Vertebrate embryos or tissues<br>• Field research<br><br>Write "N/A" if the submission does not require an ethics statement.<br><br>General guidance is provided below. Consult the submission guidelines for detailed instructions. **Make sure that all information entered here is included in the Methods section of the manuscript.**	After obtaining ethical approval from the Firat University Non-Interventional Ethics Committee (Decision no: 2023/10-31), single-rooted mandibular premolar teeth extracted from patients diagnosed with diabetes mellitus (DM) and each possessing a single round canal were collected and examined visually and radiographically. All teeth were extracted for clinical reasons unrelated to the objectives of this study, such as periodontal and orthodontic indications. Written informed consent was obtained from all patients permitting the use of their extracted teeth for research purposes. All procedures were carried out in accordance with the ethical principles outlined in the World Medical Association Declaration of Helsinki for medical research involving human tissue specimens, with strict protection of donor anonymity and confidentiality. The recruitment period for this study started on 31/10/2023 and ended on 31/10/2024. Teeth with prior root canal treatment, cracks, or caries were excluded from the study. Soft tissues and debris were removed from the collected teeth using a curette. Owing to limitations in patient records, no data were available regarding the type of diabetes or disease duration.

Powered by Editorial Manager® and ProduXion Manager® from Aries Systems Corporation

**Format for specific study types**<br><br>**Human Subject Research (involving human participants and/or tissue)**<br>• Give the name of the institutional review board or ethics committee that approved the study<br>• Include the approval number and/or a statement indicating approval of this research<br>• Indicate the form of consent obtained (written/oral) or the reason that consent was not obtained (e.g. the data were analyzed anonymously)<br><br>**Animal Research (involving vertebrate animals, embryos or tissues)**<br>• Provide the name of the Institutional Animal Care and Use Committee (IACUC) or other relevant ethics board that reviewed the study protocol, and indicate whether they approved this research or granted a formal waiver of ethical approval<br>• Include an approval number if one was obtained<br>• If the study involved *non-human primates*, add *additional details* about animal welfare and steps taken to ameliorate suffering<br>• If anesthesia, euthanasia, or any kind of animal sacrifice is part of the study, include briefly which substances and/or methods were applied<br><br>**Field Research**<br><br>Include the following details if this study involves the collection of plant, animal, or other materials from a natural setting:<br>• Field permit number<br>• Name of the institution or relevant body that granted permission	
**Data Availability**<br><br>Authors are required to make all data underlying the findings described fully available, without restriction, and from the time of publication. PLOS allows rare exceptions to address legal and ethical concerns. See the PLOS Data Policy and FAQ for detailed information.	Yes - all data are fully available without restriction

A Data Availability Statement describing where the data can be found is required at submission. Your answers to this question constitute the Data Availability Statement and **will be published in the article**, if accepted.<br><br>**Important:** Stating 'data available on request from the author' is not sufficient. If your data are only available upon request, select 'No' for the first question and explain your exceptional situation in the text box.<br><br>Do the authors confirm that all data underlying the findings described in their manuscript are fully available without restriction?	
**Describe where the data may be found in full sentences. If you are copying our sample text, replace any instances of XXX with the appropriate details.**<br><br>• If the data are **held or will be held in a public repository**, include URLs, accession numbers or DOIs. If this information will only be available after acceptance, indicate this by ticking the box below. For example: *All XXX files are available from the XXX database (accession number(s) XXX, XXX.)*.<br>• If the data are all contained **within the manuscript and/or Supporting Information files**, enter the following: *All relevant data are within the manuscript and its Supporting Information files.*<br>• If neither of these applies but you are able to provide **details of access elsewhere**, with or without limitations, please do so. For example:<br><br>*Data cannot be shared publicly because of [XXX]. Data are available from the XXX Institutional Data Access / Ethics Committee (contact via XXX) for researchers who meet the criteria for access to confidential data.*<br><br>*The data underlying the results presented in the study are available from (include the name of the third party*	All relevant data are within the manuscript and its Supporting Information files.

and contact information or URL).<br>• This text is appropriate if the data are owned by a third party and authors do not have permission to share the data.<br><br>* typeset	
Additional data availability information:	

Manuscript Click here to access/download;Manuscript;Manuscript.docx

Title: Comparative Evaluation of Root Canal Obturation Techniques and Sealers in Diabetic Dentin: An in-vitro Push-Out Bond Strength Test

Short Title: Obturation Methods in Diabetic Root Dentin

Authors: Mevlüt Sinan OCAK, DDS, PhD[1], Mehmet ESKİBAĞLAR, DDS[1], Mustafa GÜNDOĞAR, DDS, PhD[2]

1. Assistant Professor, msocak@firat.edu.tr ORCID ID: 0000-0002-3121-2116, Firat University, Faculty of Dentistry, Department of Endodontics, Elazig, Turkey

1. Associate Professor, meskibaglar@firat.edu.tr ORCID ID: 0000-0003-0183-3824, Firat University, Faculty of Dentistry, Department of Endodontics, Elazig, Turkey

2. Associate Professor, mustafagundogar@msn.com ORCID ID: 0000-0002-5795-8303, Department of Endodontics, Faculty of Dentistry, University of Medipol, Istanbul, Turkey

Corresponding Author:

Mevlüt Sinan OCAK, DDS, PhD

Firat University, Faculty of Dentistry, Department of Endodontics, Elazig, Turkey

Tel:+90 0424 237 66 01; GSM: +905337235463; e-mail: msocak@firat.edu.tr

AUTHOR CONTRIBUTIONS

Mevlüt Sinan OCAK: Methodology, investigation, data curation, writing – original draft, writing – review & editing.

Mehmet ESKİBAĞLAR: Supervision, methodology, resources, investigation, writing – review & editing.

Mustafa GÜNDOĞAR: Conceptualization, methodology, investigation, writing – review & editing.

Abstract

This study evaluated the comparative performance of different root canal obturation approaches using different sealer materials on the push-out bond strength of dentin samples obtained from patients with diabetes mellitus (DM). A total of 40 single-rooted mandibular premolars were instrumented using the Reciproc R40 system and randomly assigned to four groups (n=10 teeth each): (1) cold lateral compaction with AH Plus, (2) warm vertical compaction with AH Plus,

(3) hydraulic single-cone obturation with gutta-percha and Bioserra sealer, and (4) sealer-only technique using the Bioserra bioceramic sealer. After storage at 37°C and 100% humidity for one week, 1-mm slices were prepared from the coronal, middle, and apical thirds and subjected to push-out testing. Data were analyzed using ANOVA and Tukey's test ($p < 0.05$). Significant differences in push-out bond strength were observed among the obturation approaches employing different sealer materials ($p < 0.05$). Bioceramic sealers exhibited higher bond strengths than epoxy resin–based sealers, particularly in the middle and apical thirds. Hydraulic single-cone and bioceramic sealer-only approaches outperformed conventional techniques ($p < 0.001$), whereas no significant difference was observed between cold lateral and warm vertical compaction. Obturation approaches employing bioceramic sealers demonstrated superior bonding in diabetic dentin, suggesting potential advantages over conventional methods within the context of diabetic dentin.

Keywords: Bioceramic Sealer; Dentin Adhesion; Diabetes Mellitus; Root Canal Obturation; Push-Out Bond Strength

INTRODUCTION

Diabetes mellitus (DM) is a complex multisystemic metabolic disorder projected to affect nearly 853 million people by 2050. Currently, more than 589 million adults aged 20–79 years live with DM worldwide, reflecting a significant global health burden.[1] DM is characterized by persistent hyperglycemia and arises from a combination of genetic and environmental factors.[2] It exerts systemic effects on multiple organs and tissues, including the oral cavity.[3] Previous studies have shown that poor glycemic control can negatively affect the dental pulp, connective tissues, and cellular structures, thereby compromising oral health.[4-6] Dentin, a mineralized tissue composed of approximately 70% inorganic material, 20% organic matrix, and 10% water, is highly relevant for endodontic treatment.[7] DM alters the trace element composition of dentin—particularly strontium, magnesium, zinc, and selenium—within the hydroxyapatite structure. These changes may affect the crystallinity and physicochemical characteristics of the mineral phase, potentially weakening the biomechanical properties of dentin, reducing

microhardness, lowering fracture resistance, and impairing adhesion capacity of root canal filling materials.[8,9]

Given these substrate alterations, the interaction between dentin and obturation materials may be material-dependent. Calcium silicate–based bioceramic sealers release calcium ions and promote biomineralization at the sealer–dentin interface, resulting in the formation of hydroxyapatite-like interfacial structures and chemical interactions with the inorganic phase of dentin.[10-12] Therefore, in mineral-altered diabetic dentin, it may be hypothesized that bioceramic sealers may establish enhanced substrate interactions through ionic exchange and interfacial mineral deposition compared with epoxy resin–based sealers, which rely predominantly on micromechanical retention and limited chemical bonding.[12] This potential difference in the bonding mechanism may be particularly relevant in structurally compromised dentin affected by chronic hyperglycemia.[9,13]

Root canal obturation aims to achieve a fluid-tight seal along the root canal system, commonly using gutta-percha (GP) in combination with a sealer. Traditional techniques, such as cold lateral compaction, are still widely used but have limitations, including insufficient adaptation to canal walls and potential root fractures due to microcrack formation. Warm vertical compaction improves adaptation but remains technique sensitive.[14,15] In contrast, newly developed calcium silicate–based bioceramic sealers, such as Bioserra, which is a premixed sealer containing tricalcium silicate and dicalcium silicate as bioactive components, together with calcium aluminate, calcium aluminum oxide, tricalcium aluminate, and tantalum as radiopacifiers, have demonstrated superior push-out bond strength compared to resin-based sealers in healthy dentin.[16] However, their performance in structurally compromised diabetic dentin is unclear.

One of the main objectives of root canal obturation is to reinforce the root dentin and increase its resistance to fracture.[17] It has been suggested that materials that adhere to the dentin surface can contribute to strengthening the remaining tooth structure.[18] The push-out bond strength test is widely used to evaluate the adhesion between obturation materials and canal walls because

of its simplicity and reproducibility.[19] This test reflects a combination of frictional forces, molecular interactions, and chemical bonding between the materials and dentinal walls.[20]

A review of the available literature indicates that, although several studies have assessed the bonding ability of individual materials, such as mineral trioxide aggregate (MTA) or specific sealers, in diabetic dentin,[13,21] limited evidence exists regarding how different obturation techniques influence bond strength in this altered substrate. Most previous investigations have focused primarily on material properties rather than on technique-dependent variables. Furthermore, the current literature highlights the limited and heterogeneous nature of data concerning the mechanical performance of diabetic dentin.[9] To our knowledge, no study has directly compared multiple root canal obturation approaches in diabetic dentin under standardized conditions while isolating the intrinsic bonding potential of sealers. Therefore, this study aimed to evaluate the influence of various obturation techniques and sealer types on the push-out bond strength in dentin derived from diabetic patients. The present study was not intended to compare diabetic and healthy dentin. Instead, it focused exclusively on dentin derived from diabetic patients to evaluate the relative performance of different obturation techniques under clinically compromised conditions, allowing assessment of intragroup differences without additional confounding factors.

Therefore, this study aimed to compare the push-out bond strength of different root canal obturation approaches using different sealer materials in teeth obtained from patients with diabetes mellitus. A sealer-only group was included to isolate and assess the direct interaction between the sealer and dentin, independent of the influence of the gutta-percha. The null hypothesis (H_0) was that there would be no significant differences in the push-out bond strength among the tested obturation techniques.

MATERIALS AND METHODS

Sample Selection

The required sample size was calculated using GPower software (GPower 3.1.7, Windows, Düsseldorf, Germany) to obtain eight specimens per group. To increase the robustness of the

analysis and compensate for potential specimen loss, the final sample size was increased to 10 specimens per group (40 teeth).

After obtaining ethical approval from the Firat University Non-Interventional Ethics Committee (Decision no: 2023/10-31), single-rooted mandibular premolar teeth extracted from patients diagnosed with diabetes mellitus (DM) and each possessing a single round canal were collected and examined visually and radiographically. All teeth were extracted for clinical reasons unrelated to the objectives of this study, such as periodontal and orthodontic indications. Written informed consent was obtained from all patients permitting the use of their extracted teeth for research purposes. All procedures were carried out in accordance with the ethical principles outlined in the World Medical Association Declaration of Helsinki for medical research involving human tissue specimens, with strict protection of donor anonymity and confidentiality. The recruitment period for this study started on 31/10/2023 and ended on 31/10/2024. Teeth with prior root canal treatment, cracks, or caries were excluded from the study. Soft tissues and debris were removed from the collected teeth using a curette. Owing to limitations in patient records, no data were available regarding the type of diabetes or disease duration. Only patients with documented diabetes mellitus and HbA1c levels ≥ 6.5% obtained from their medical records at the time of tooth extraction were included in the study.[1]

To standardize the root lengths, the roots were sectioned under water cooling with a diamond disc to obtain a 12 millimeters (mm) segment from the apex. Canal patency was confirmed using a #15 K-file (Dentsply/Maillefer, Ballaigues, Switzerland). The root canals were then instrumented using the Reciproc system (VDW, Munich, Germany) up to size R40. During the shaping procedure, a total of 10 mL of 2.5% sodium hypochlorite (NaOCl) was used per canal, delivered incrementally using a flexible, side-vented irrigation needle. After instrumentation, the smear layer was removed using 1 mL of 17% ethylenediaminetetraacetic acid (EDTA) for 1 min. Final irrigation was performed with 2 mL of distilled water, and the canals were dried with sterile paper points.

Root Canal Obturation

Randomization was performed at the tooth level using a simple random allocation method prior to the sectioning. Ten teeth were allocated to each experimental group. Each tooth was assigned to a single group to ensure balanced distribution and to prevent intra-tooth correlation across groups. All obturation procedures were performed by a single, experienced operator using various techniques.

•In Group 1, cold lateral compaction was performed using an AH Plus sealer (Dentsply DeTrey GmbH, Konstanz, Germany).

•In Group 2, warm vertical compaction was performed using the AH Plus sealer and the Elements Free cordless obturation system (SybronEndo/Kerr Endodontics, Orange, CA, USA).

•In Group 3, single-cone obturation was performed using gutta-percha and Bioserra sealer (Meta Biomed, Dongsaeng Myeong 1-ro, Korea).

•In Group 4, the root canals were filled exclusively with the Bioserra sealer without the use of gutta-percha to evaluate the intrinsic bonding ability of the sealer to root dentin.

To ensure complete setting of the root canal sealers, the specimens were stored at 37°C and 100% humidity for one week. After obturation, standardized slices of 1 mm thickness were obtained from the coronal, middle, and apical thirds (at 2.5, 5, and 8 mm from the apex, respectively) using a water-cooled diamond saw (Isomet, Buehler Ltd., USA).

Push-Out Bond Strength Test

The 120 slices were subjected to a push-out test using a universal testing machine (Instron Industrial Products, Norwood, MA, USA) at a crosshead speed of 1 mm/min in the apico-coronal direction (Figure 1). Different plungers were used for the coronal, middle, and apical sections to ensure a proper fit within the canal space, avoiding contact with the surrounding dentin. Each specimen was positioned with its apical side facing the plunger to minimize stress on the dentin.

Figure 1: Sample preparation and push-out testing of root slices after obturation with different techniques and materials.

The force required to dislodge the filling material (in kilonewtons (kN)) was converted to stress (in megapascals (MPa)) [22]. The upper and lower diameters of each specimen were measured individually using the following formula:

MPa = F / SL,

where SL = sealer adhesion area = $\pi(R + r)g$,

$\pi = 3.14$, R = coronal canal radius (mm), r = apical canal radius (mm), and g = slice thickness (mm).

Dislodgement of the filling was confirmed by a sudden drop in the load–displacement curve. The push-out bond strength was calculated for each root slice. For statistical analysis, the mean push-out bond strength values obtained from the coronal, middle, and apical slices of each tooth were calculated.

Data Analysis

Statistical analyses were performed using IBM SPSS Statistics for Windows, Version 23.0 (SPSS Inc., Chicago, IL, USA). For statistical analysis, the mean push-out bond strength value per tooth was considered as the statistical unit. The normality of the data was assessed using the Kolmogorov–Smirnov and Shapiro–Wilk tests, and the homogeneity of variances was verified using Levene's test. For normally distributed data with equal variances, one-way ANOVA was used to compare the push-out bond strength values, followed by Tukey's HSD post hoc test for pairwise comparisons. The results are presented as mean ± standard deviation, with a significance level set at $p < 0.05$.

RESULTS

No statistically significant differences were observed between the Cold Lateral and Warm Vertical groups in the coronal, middle, or apical thirds ($p > 0.05$; Table 1). In contrast, both the hydraulic single cone and bioceramic sealer-only groups exhibited significantly higher push-out bond strength values in all root regions compared with the conventional techniques ($p < 0.001$; Table 1). A significant difference was also detected between the hydraulic single cone

and bioceramic sealer-only groups in the coronal region, with higher values observed in the bioceramic sealer-only group ($p < 0.05$; Table 1).

Table 1. Push-out bond strength (MPa) according to obturation technique and root region (mean ± SD).

Coronal					Middle					Apical				
	N	Mean	Std. Deviation	P*		N	Mean	Std. Deviation	P*		N	Mean	Std. Deviation	P*
Cold Lateral	10	3.716	0.678	<0.001	Cold Lateral	10	4.731	0.545	<0.001	Cold Lateral	10	4.469	0.746	<0.001
Warm Vertical	10	3.249	0.701		Warm Vertical	10	5.742	0.889		Warm Vertical	10	5.444	1.016	
Hydraulic ^{a1,b1}	10	5.086	0.655		Hydraulic ^{a1,b1}	10	9.246	0.938		Hydraulic ^{a1,b1}	10	8.787	1.097	
Bioceramic ^{a2,b2,c}	10	9.140	0.985		Bioceramic ^{a,b,c}	10	13.123	1.408		Bioceramic ^{a,b,c}	10	11.101	1.332	

* All groups were analyzed using one-way ANOVA followed by Tukey's HSD post hoc test. For each root region (coronal, middle, and apical), different lowercase letters (a, b, c) indicate statistically significant differences among obturation techniques ($p < 0.05$). Numerical superscripts (1, 2) indicate statistically significant differences among root regions within the same obturation technique ($p < 0.05$).

In the intragroup analysis of the bioceramic sealer-only group, bond strength values in the middle and apical thirds were significantly higher than those in the coronal third ($p < 0.001$). Moreover, a significant difference was observed between the middle and apical thirds ($p < 0.001$; Table 2). A similar pattern was observed in the hydraulic single cone group, with significantly higher values in the middle and apical thirds than in the coronal third ($p < 0.001$). This trend was also observed in the Warm Vertical and Cold Lateral groups, although with a lower magnitude and statistical significance ($p < 0.05$; Table 2).

Table 2. Regional comparison of push-out bond strength (MPa) within each obturation technique (mean ± SD).

Cold Lateral					Warm Vertical					Hydraulic					Bioceramic				
	N	Mean	Std. Deviation	P*		N	Mean	Std. Deviation	P*		N	Mean	Std. Deviation	P*		N	Mean	Std. Deviation	P*
Coronal	10	3.716	0.678	<0.001	Coronal	10	3.249	0.701	<0.001	Coronal	10	5.086	0.655	<0.001	Coronal	10	9.140	0.985	<0.001
Middle [a1]	10	4.731	0.545		Middle [a1]	10	5.742	0.889		Middle [a1]	10	9.246	0.938		Middle [a1]	10	13.123	1.408	
Apical [a2]	10	4.469	0.746		Apical [a2]	10	5.444	1.016		Apical [a2]	10	8.787	1.097		Apical [a2,b]	10	11.101	1.332	

*All groups were analyzed using one-way ANOVA followed by Tukey's HSD post hoc test. Within each obturation technique, different lowercase letters indicate statistically significant differences among root regions. Numerical superscripts indicate statistically significant differences among obturation techniques within the same root region ($p < 0.05$).

When the obturation techniques were compared across all root regions, both the bioceramic sealer-only and hydraulic single cone groups showed significantly higher overall push-out bond strengths than the conventional groups ($p < 0.001$; Table 3). Bioceramic sealer-only group demonstrated the highest overall mean bond strength, followed in descending order by the Hydraulic Single Cone, Warm Vertical, and Cold Lateral groups.

Table 3. Overall comparison of push-out bond strength (MPa) among obturation techniques (mean ± SD).

	N	Mean	Std. Deviation	P*
Cold Lateral	10	4.305	0.774	<0.001
Warm Vertical	10	4.811	1.413	
Hydraulic [a,b]	10	7.706	2.090	
Bioceramic [a,b,c]	10	11.121	2.050	

*Statistical analysis was performed using one-way ANOVA followed by Tukey's HSD post hoc test. Different lowercase letters (a, b, c) indicate statistically significant differences among obturation techniques ($p < 0.05$).

DISCUSSION

This study investigated the influence of various root canal obturation techniques and sealer types on the push-out bond strength in dentin derived from diabetic patients. Chronic hyperglycemia in diabetes promotes the accumulation of advanced glycation end-products (AGEs), which can disrupt collagen integrity.[9] Diabetes has been associated with an increased susceptibility to vertical root fracture,[23] which may indirectly reflect alterations in dentin mechanical behavior. These changes are generally associated with impaired adhesion of resin-based materials, as hybrid layer formation depends on collagen integrity.[24,25] Therefore, AGE-induced alterations in dentin collagen could potentially influence the bonding performance of epoxy resin–based sealers.[21,26] In contrast, calcium silicate–based bioceramic sealers rely less on collagen-dependent micromechanical retention and more on ionic exchange and biomineralization with the inorganic phase of dentin.[10-12] This difference in the bonding mechanism may explain the superior performance of the bioceramic materials observed in the present study. However, limited data exists regarding how such alterations affect the adhesion of root canal sealers in diabetic dentin.[13,27] Although previous studies have

demonstrated the bonding potential of bioceramic sealers to healthy dentin through chemical interactions,[11,28] the present study uniquely evaluated their performance in biochemically compromised dentin under clinically relevant conditions.

The results demonstrated that both the obturation technique and type of sealer had a significant impact on push-out bond strength. Groups utilizing bioceramic sealers exhibited consistently higher bond strength across all root sections than those utilizing techniques ($p < 0.001$), suggesting that the chemical bonding capacity and bioactivity of calcium silicate–based materials may offer distinct advantages in diabetic dentin.[10]

In the coronal region, the bioceramic sealer-only group demonstrated significantly higher bond strength than the hydraulic single cone group ($p < 0.001$; Table 1). This difference may be attributed to the more homogeneous sealer mass and greater interface adaptation achieved with the bioceramic technique, which enhances chemical bonding and reduces voids. In support of this, previous studies have shown that calcium silicate–based sealers exhibit superior dentinal tubule penetration and promote biomineralization owing to their hydrophilic and bioactive properties, which reinforce interfacial bonding over time.[10,11,29]

Within the Bioceramic sealer-only group, the bond strength values in the middle and apical thirds were significantly higher than those in the coronal region ($p < 0.001$; Table 2), which is consistent with previous studies reporting increased push-out bond strength toward the middle and apical root thirds for calcium silicate–based sealers.[30.31] This finding may be related to regional differences in the dentin morphology and canal geometry. Although apical dentin generally presents with narrower dentinal tubules, the reduced canal diameter and increased confinement of the filling material may enhance frictional resistance and sealer adaptation in the apical sections.[12] Additionally, diabetes-induced alterations in the dentin structure, including changes in collagen integrity and mineral composition associated with AGEs, may influence bonding behavior in a region-dependent manner.[13] However, these proposed explanations remain hypothetical, as the present study did not directly assess the regional structural or biochemical differences in diabetic dentin.

In the hydraulic single cone group, bond strength values in the middle and apical thirds were significantly higher than those in the coronal third ($p < 0.05$; Table 2). Conversely, Cold Lateral and Warm Vertical compaction demonstrated significantly lower bond strengths than bioceramic-based approaches ($p < 0.05$; Table 3). These findings indicate that resin-based sealers may offer limited adhesion to structurally compromised diabetic dentin, likely because they rely on mechanical interlocking rather than chemical bonding.[32]

Bioceramic sealers demonstrated the highest bond strength values in the middle and apical thirds, further confirming their superior adhesion characteristics in root regions with narrower tubules and a higher surface area ($p < 0.001$; Table 2). These results highlight the potential clinical relevance of calcium silicate–based sealers in diabetic dentin, where microstructural and biochemical changes may compromise the effectiveness of conventional obturation systems.

Although the standard deviation values did not exceed the mean values, a relatively greater dispersion was observed in certain groups, particularly those involving bioceramic sealers. This variability may reflect the heterogeneity of dentin substrate quality among individuals with diabetes.[9,13] Chronic hyperglycemia does not affect dentin uniformly; variations in disease duration and metabolic history may lead to heterogeneous structural and biochemical alterations.[9] Such changes can influence the mineral composition, collagen integrity, and biomechanical behavior of dentin,[13] which may partially account for the variability observed in the push-out bond strength measurements in the present study.

This study has several limitations that should be considered when interpreting the findings. First, the specimens were collected from diabetic patients of variable ages and disease durations, which may have introduced heterogeneity in dentin structure and mechanical behavior.[9,13] Second, the push-out bond strength test was conducted in vitro, lacking physiological conditions such as masticatory loading, thermal cycling, and salivary interactions, which may affect long-term performance.[33] Third, only single-rooted canals with standardized anatomy were used, and variations in canal morphology or irrigation protocols were not

explored. Finally, although previous studies have included non-diabetic control groups to characterize diabetes-related dentin changes,[13,27] the present study focused exclusively on diabetic samples to isolate the effects of the obturation techniques. These limitations warrant caution when generalizing the results to broader clinical scenarios.

Despite these limitations, the present study provides valuable insights into the bonding behavior of root canal obturation materials in a clinically relevant diabetic dentin model, addressing a significant gap in the endodontic literature. Future studies should explore the biochemical mechanisms of sealer–dentin interactions in diabetes, including the role of advanced glycation end-products and collagen alterations. The absence of a non-diabetic control group should be considered when interpreting the results, as the primary objective of the present study was not to compare diabetic and non-diabetic dentin but to evaluate the relative performance of different obturation techniques within a diabetic population. Aging protocols that simulate intraoral conditions (e.g., thermocycling and mechanical loading) are also required to assess long-term material performance. Although diabetes was confirmed using HbA1c values of $\geq$ 6.5%, detailed information regarding diabetes type, disease duration, and long-term metabolic control was not available, which should be considered when interpreting these results.

From a clinical standpoint, the interaction between dentin substrate quality and sealer characteristics may be particularly relevant in patients with systemic conditions, such as diabetes. Although the present findings are based on in vitro conditions, the improved bonding performance of bioceramic sealers in diabetic dentin suggests that material selection may play a critical role in endodontic treatment outcomes in medically compromised patients. The findings are specific to diabetic dentin and should not be extrapolated to healthy substrates; however, comparative evaluation of obturation techniques within compromised dentin remains clinically relevant.

CONCLUSIONS

Within the limitations of this in vitro study, both the obturation technique and the type of sealer significantly influenced bond strength in diabetic dentin. Bioceramic-based obturation

exhibited superior adhesion compared to conventional methods, underscoring its potential clinical relevance in the endodontic treatment of diabetic dentin.

ACKNOWLEDGEMENTS

None.

AUTHOR CONTRIBUTIONS

Mevlüt Sinan OCAK: Methodology, investigation, data curation, writing – original draft, writing – review & editing.

Mehmet ESKİBAĞLAR: Supervision, methodology, resources, investigation, funding acquisition, resources, writing – review & editing.

Mustafa GÜNDOĞAR: Conceptualization, methodology, investigation, writing – review & editing.

Statements and Declarations

Not applicable

Consent to participate

Not applicable

Consent for publication

Not applicable

Declaration of conflicting interest

The authors declare no potential conflicts of interest with respect to the research, authorship, and/or publication of this article.

References

1. Schwarz P. International Diabetes Federation: IDF Diabetes Atlas 2025. 10 Nov 2025 [Cited 15 Dec 2025]. Available from: https://diabetesatlas.org/resources/idf-diabetes-atlas-2025

2. L. Tierney, S. McPhee, M. Papadakis, Current Medical Diagnosis and Treatment. International ed. New York: Lange Medical Books,McGraw-Hill; 2002.

3. Graves DT, Liu R, Oates TW. Diabetes-enhanced inflammation and apoptosis: impact on periodontal pathosis. *Periodontol 2000*. 2007;45:128-37. doi: 10.1111/j.1600-0757.2007.00219.x

4. Bender IB, Bender AB. Diabetes mellitus and the dental pulp. *J Endod*. 2003;29:383-9. doi: 10.1097/00004770-200306000-00001

5. Catanzaro O, Dziubecki D, Lauria LC, et al. Diabetes and its effects on dental pulp. *J Oral Sci.* 2006;48:195-9. doi: 10.2334/josnusd.48.195

6. Lima SM, Grisi DC, Kogawa EM, et al. Diabetes mellitus and inflammatory pulpal and periapical disease: a review. *Int Endod J*. 2013;46:700-9. doi: 10.1111/iej.12072

7. Goldberg M, Kulkarni AB, Young M, et al. Dentin: structure, composition and mineralization. *Front Biosci (Elite Ed)*. 2011;3:711-35. doi: 10.2741/e281

8. Saghiri MA, Vakhnovetsky J, Samadi E, et al. Effects of Diabetes on Elemental Levels and Nanostructure of Root Canal Dentin. *J Endod*. 2023;49:1169-1175. doi: 10.1016/j.joen.2023.07.002

9. Hwang KC, Choi JJE, Hussaini HM, et al. Effect of diabetes and hyperglycaemia on the physical and mechanical properties of dentine: a systematic review. *Clin Oral Investig*. 2025;29:55. doi: 10.1007/s00784-025-06151-5

10. Shieh K, Yang J, Zhu EH, et al. Dentinal Tubule Penetrability and Bond Strength of Two Novel Calcium Silicate-Based Root Canal Sealers. *Materials (Basel)*. 2023;16:3309. doi: 10.3390/ma16093309

11. Radulica N, Sanz JL, Lozano A. Dentin Bond Strength of Calcium Silicate-Based Materials: A Systematic Review of In Vitro Studies. *Applied Sciences*. 2024;14:104. doi: 10.3390/app14010104

12. Chisnoiu RM, Moldovan M, Prodan D, et al. In-Vitro Comparative Adhesion Evaluation of Bioceramic and Dual-Cure Resin Endodontic Sealers Using SEM, AFM, Push-Out and FTIR. *Applied Sciences*. 2021;11:4454. doi: 10.3390/app11104454

13. Saghiri MA, Karamifar K, Fakharzadeh A, et al. Effect of Diabetes on Tubular Density and Push-out Bond Strength of Mineral Trioxide Aggregate to Dentin. *J Endod*. 2020;46:1584-1591. doi: 10.1016/j.joen.2020.07.025

14. Evans JT, Simon JH. Evaluation of the apical seal produced by injected thermoplasticized Gutta-percha in the absence of smear layer and root canal sealer. *J Endod*. 1986;12:100-7. PMID: 3517216.

15. Shemesh H, Wesselink PR, Wu MK. Incidence of dentinal defects after root canal filling procedures. *Int Endod J*. 2010;43:995-1000. doi: 10.1111/j.1365-2591.2010.01740.x

16. Fundaoğlu Küçükekenci F. The effect of different acidic irrigation solutions on the pushout bond strength of root canal filling. *J Dent Res Dent Clin Dent Prospects*. 2023;17:18-22. doi: 10.34172/joddd.2023.36920

17. Ulusoy OI, Genç O, Arslan S, et al. Fracture resistance of roots obturated with three different materials. *Oral Surg Oral Med Oral Pathol Oral Radiol Endod*. 2007;104:705-8. doi: 10.1016/j.tripleo.2007.07.013

18. Johnson ME, Stewart GP, Nielsen CJ, et al. Evaluation of root reinforcement of endodontically treated teeth. *Oral Surg Oral Med Oral Pathol Oral Radiol Endod*. 2000;90:360-4. doi: 10.1067/moe.2000.108951

19. Wang JS, Bai W, Wang Y, et al. Effect of different dentin moisture on the push-out strength of bioceramic root canal sealer. *J Dent Sci*. 2023;18:129-134. doi: 10.1016/j.jds.2022.06.025

20. Bohn S, Ilie N. Wetting behaviour of silicone- and resin-based root canal sealers. *Int Endod J*. 2014;47:542-9. doi: 10.1111/iej.12184

21. Oncu A, Ozgur E, Efeoglu S, et al. Evaluation of the effects of diabetes on tooth radiodensity, bond strength of root canal sealers to dentin, and interfacial elemental distribution. *BMC Oral Health*. 2025;25:1780. doi: 10.1186/s12903-025-07155-z

22. Costa JA, Rached-Júnior FA, Souza-Gabriel AE, et al. Push-out strength of methacrylate resin-based sealers to root canal walls. *Int Endod J*. 2010;43:698-706. doi: 10.1111/j.1365-2591.2010.01766.x

23. PradeepKumar AR, JothiLatha S, Durvasulu A, et al. Impact of Type 2 Diabetes Mellitus on the Occurrence of Vertical Root Fracture: A Case Control Study. *J Endod*. 2024;50:450-455.e1. doi: 10.1016/ j.joen.2024.01.006

24. H S Delgado A, Belmar Da Costa M, Polido MC, et al. Collagen-depletion strategies in dentin as alternatives to the hybrid layer concept and their effect on bond strength: a systematic review. *Sci Rep*. 2022;12:13028. doi: 10.1038/s41598-022-17371-0

25. Breschi L, Maravic T, Cunha SR, et al. Dentin bonding systems: From dentin collagen structure to bond preservation and clinical applications. *Dent Mater*. 2018;34:78-96. doi: 10.1016/j.dental.2017.11.005

26. Miura J, Nishikawa K, Kubo M, et al. Accumulation of advanced glycation end-products in human dentine. *Arch Oral Biol*. 2014;59:119-24. doi: 10.1016/j.archoralbio.2013.10.012

27. Arslandaş Dinçtürk B, Şahin Mantı A, Kedici Alp C, et al. Improving the Push-Out Bond Strength of Fiber Posts in Diabetic Dentin: The Role of Chlorexidine Irrigation and Resin Cements. *J Funct Biomater*. 2024;16:4. doi: 10.3390/jfb16010004

28. Viapiana R, Flumignan DL, Guerreiro-Tanomaru JM, et al. Physicochemical and mechanical properties of zirconium oxide and niobium oxide modified Portland cement-based experimental endodontic sealers. *Int Endod J*. 2014;47:437-48. doi: 10.1111/iej.12167

29. Mann NS, Mann NK, Kapur R. Evaluating the penetration efficacy of calcium silicate-based bioceramic sealers into dentinal tubules with cold lateral compaction technique using confocal laser scanning microscopy: An in vitro study. *J Conserv Dent Endod*. 2025;28:150-154. doi: 10.4103/JCDE.JCDE_754_24

30. Cimpean SI, Burtea ALC, Chiorean RS, et al. Evaluation of Bond Strength of Four Different Root Canal Sealers. *Materials (Basel)*. 2022;15:4966. doi: 10.3390/ma15144966

31. Khoury S, Aranda-Verdú S, Casino-Alegre A, et al. Comparison of the push-out bond strength of two hydraulic calcium silicate-based endodontic sealers and an epoxy resin-based sealer. *J Clin Exp Dent*. 2023;15:e804-e809. doi: 10.4317/jced.60920

32. Araujo VLC, Cruvinel PB, Palma-Dibb RG, et al. In vitro bond strength of an epoxy resin-based root canal sealer to root dentin irradiated with high-power lasers and adhesive interface analyses. *Lasers Med Sci*. 2018;33:271-277. doi: 10.1007/s10103-017-2362-z

33. Chen WP, Chen YY, Huang SH, et al. Limitations of push-out test in bond strength measurement. *J Endod*. 2013;39:283-7. doi: 10.1016/j.joen.2012.11.002.

Click here to access/download;Figure;Figure-1.jpeg

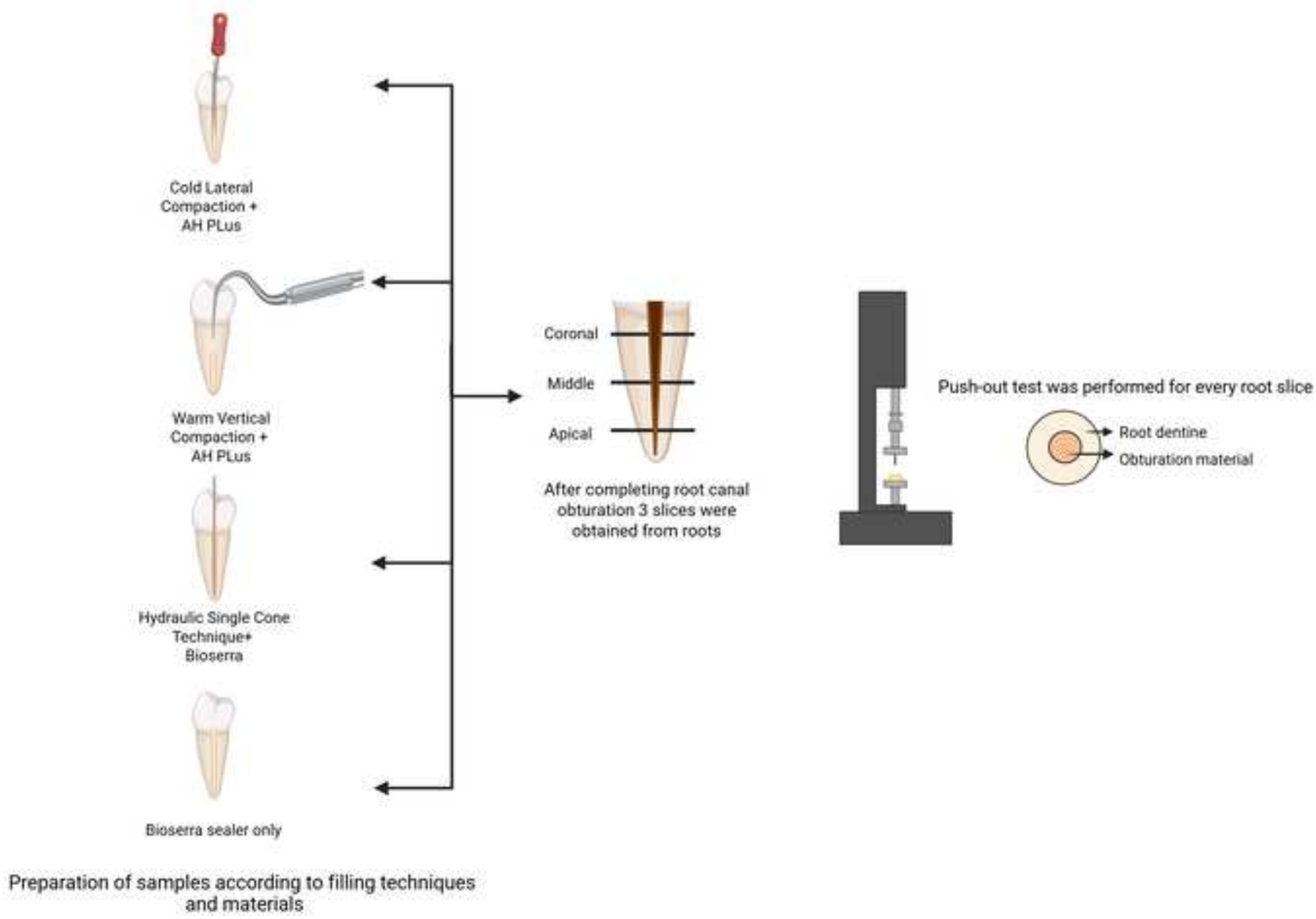

Raw Data

Click here to access/download
Supporting Information
Push- out veriler.xlsx
