# Supplementary material for: Comparative evaluation of root canal obturation techniques and sealers in Diabetic Dentin: An in-vitro push-out bond strength test
Source: PLoS One. 2026 Jul 17;21(7):e0353975. doi: 10.1371/journal.pone.0353975 (PMC13378988; doi:10.1371/journal.pone.0353975)
Supplement: Attachment — Submitted filename: Response to Reviewer - 2.docx [file pone.0353975.s004.docx]

Response to Editor:

Dear Editor,

Thank you for your message. We confirm that the Supporting Information file “S1 Data.xlsx” contains the complete minimal dataset required to reproduce the results of our study.

This dataset includes all individual raw measurements used to calculate the reported means, standard deviations, and statistical analyses. Specifically, it contains the push-out bond strength values (MPa) for each specimen, including group allocation and root region (coronal, middle, apical).

No additional data were used beyond those provided in this file to generate the results presented in the manuscript.

We confirm that the dataset is sufficient to fully reproduce the findings of the study.

Kind regards,

Dr. Mevlüt Sinan Ocak
