# Supplementary material for: Comparative evaluation of root canal obturation techniques and sealers in Diabetic Dentin: An in-vitro push-out bond strength test
Source: PLoS One. 2026 Jul 17;21(7):e0353975. doi: 10.1371/journal.pone.0353975 (PMC13378988; doi:10.1371/journal.pone.0353975)
Supplement: Peer review history

# Peer review: Decision Letter 0

**DOI:** 10.1371/journal.pone.0353975.r001

**Authors:** Mohmed Isaqali Karobari

12 Apr 2026

PONE-D-26-12466

Comparative Evaluation of Root Canal Obturation Techniques and Sealers in Diabetic Dentin: An in-vitro Push-Out Bond Strength Test

PLOS One

Dear Dr. OCAK,

Thank you for submitting your manuscript to PLOS ONE. After careful consideration, we feel that it has merit but does not fully meet PLOS ONE’s publication criteria as it currently stands. Therefore, we invite you to submit a revised version of the manuscript that addresses the points raised during the review process.

==============================

Dear Authors,

Please carefully read all the reviewers' comments and address them accordingly, making the necessary changes in the revised manuscript.

Best regards and keep well

==============================

Please submit your revised manuscript by May 27 2026 11:59PM. If you will need more time than this to complete your revisions, please reply to this message or contact the journal office at plosone@plos.org. When you're ready to submit your revision, log on to https://www.editorialmanager.com/pone/ and select the 'Submissions Needing Revision' folder to locate your manuscript file.

Please include the following items when submitting your revised manuscript:

- A letter that responds to each point raised by the academic editor and reviewer(s). You should upload this letter as a separate file labeled 'Response to Reviewers'.
- A marked-up copy of your manuscript that highlights changes made to the original version. You should upload this as a separate file labeled 'Revised Manuscript with Track Changes'.
- An unmarked version of your revised paper without tracked changes. You should upload this as a separate file labeled 'Manuscript'.

If you would like to make changes to your financial disclosure, please include your updated statement in your cover letter. Guidelines for resubmitting your figure files are available below the reviewer comments at the end of this letter.

If applicable, we recommend that you deposit your laboratory protocols in protocols.io to enhance the reproducibility of your results. Protocols.io assigns your protocol its own identifier (DOI) so that it can be cited independently in the future. For instructions see: https://journals.plos.org/plosone/s/submission-guidelines#loc-laboratory-protocols. Additionally, PLOS ONE offers an option for publishing peer-reviewed Lab Protocol articles, which describe protocols hosted on protocols.io. Read more information on sharing protocols at https://plos.org/protocols?utm_medium=editorial-email&utm_source=authorletters&utm_campaign=protocols.

As the corresponding author, your ORCID iD is verified in the submission system and will appear in the published article. PLOS supports the use of ORCID, and we encourage all coauthors to register for an ORCID iD and use it as well. Please encourage your coauthors to verify their ORCID iD within the submission system before final acceptance, as unverified ORCID iDs will not appear in the published article. *Only*  the individual author can complete the verification step; PLOS staff *cannot*  verify ORCID iDs on behalf of authors.

We look forward to receiving your revised manuscript.

Kind regards,

Mohmed Isaqali Karobari, BDS, MScD.Endo, Ph.D. Endo, FDS, FPFA, FICD, MFDS

Academic Editor

PLOS One

Journal Requirements:

When submitting your revision, we need you to address these additional requirements.

1.Please ensure that your manuscript meets PLOS ONE's style requirements, including those for file naming. The PLOS ONE style templates can be found at

https://journals.plos.org/plosone/s/file?id=wjVg/PLOSOne_formatting_sample_main_body.pdf and

https://journals.plos.org/plosone/s/file?id=ba62/PLOSOne_formatting_sample_title_authors_affiliations.pdf

2. Thank you for providing your underlying data as Supporting Information.

We note that the data set contains text or data that is not in English. Please note that PLOS is an English-language publisher, so we require data sets to be provided in English as well. Please upload an English-language version of your data set.

This will also allow us to determine if your data follows PLOS standards per our Data Availability policy here: https://journals.plos.org/plosone/s/data-availability.

3. Please include captions for your Supporting Information files at the end of your manuscript, and update any in-text citations to match accordingly. Please see our Supporting Information guidelines for more information: http://journals.plos.org/plosone/s/supporting-information.

4. If the reviewer comments include a recommendation to cite specific previously published works, please review and evaluate these publications to determine whether they are relevant and should be cited. There is no requirement to cite these works unless the editor has indicated otherwise.

5. Please review your reference list to ensure that it is complete and correct. If you have cited papers that have been retracted, please include the rationale for doing so in the manuscript text, or remove these references and replace them with relevant current references. Any changes to the reference list should be mentioned in the rebuttal letter that accompanies your revised manuscript. If you need to cite a retracted article, indicate the article’s retracted status in the References list and also include a citation and full reference for the retraction notice.

Additional Editor Comments:

Dear Authors,

Please carefully read all the reviewers' comments and address them accordingly, making the necessary changes in the revised manuscript.

Best regards and keep well

[Note: HTML markup is below. Please do not edit.]

Reviewer's Responses to Questions

**Comments to the Author**

1. Is the manuscript technically sound, and do the data support the conclusions?

The manuscript must describe a technically sound piece of scientific research with data that supports the conclusions. Experiments must have been conducted rigorously, with appropriate controls, replication, and sample sizes. The conclusions must be drawn appropriately based on the data presented.

Reviewer #1: Yes

Reviewer #2: Partly

**********

2. Has the statistical analysis been performed appropriately and rigorously?

Reviewer #1: Yes

Reviewer #2: Yes

**********

3. Have the authors made all data underlying the findings in their manuscript fully available?

The PLOS Data policy requires authors to make all data underlying the findings described in their manuscript fully available without restriction, with rare exception (please refer to the Data Availability Statement in the manuscript PDF file). The data should be provided as part of the manuscript or its supporting information, or deposited to a public repository. For example, in addition to summary statistics, the data points behind means, medians and variance measures should be available. If there are restrictions on publicly sharing data—e.g. participant privacy or use of data from a third party—those must be specified.

Reviewer #1: Yes

Reviewer #2: Yes

**********

4. Is the manuscript presented in an intelligible fashion and written in standard English?

PLOS ONE does not copyedit accepted manuscripts, so the language in submitted articles must be clear, correct, and unambiguous. Any typographical or grammatical errors should be corrected at revision, so please note any specific errors here.

Reviewer #1: Yes

Reviewer #2: Yes

**********

5. Review Comments to the Author

Please use the space provided to explain your answers to the questions above. You may also include additional comments for the author, including concerns about dual publication, research ethics, or publication ethics. (Please upload your review as an attachment if it exceeds 20,000 characters)

Reviewer #1: kindly do a english proof reading ,add recent article in citation , mention the limitation before the conclusion is mentioned

was age of patients taken into consideration?

was the gender noted when the teeth were selected for extraction?

would age and gender of the patient influence the results obtained ?

eas it checked

Reviewer #2: The groups have different techniques and different materials (sealers) used. This adds to the confounding factor to the statistical analysis. It is like comparing oranges with apples.

If for first and second group, the bioceramic sealer was used, then it would be unbiased comparison between the obturation techniques, according to the aim of the study.

**********

6. PLOS authors have the option to publish the peer review history of their article (what does this mean?). If published, this will include your full peer review and any attached files.

If you choose “no”, your identity will remain anonymous but your review may still be made public.

**Do you want your identity to be public for this peer review?**  For information about this choice, including consent withdrawal, please see our Privacy Policy.

Reviewer #1: No

Reviewer #2: No

**********

[NOTE: If reviewer comments were submitted as an attachment file, they will be attached to this email and accessible via the submission site. Please log into your account, locate the manuscript record, and check for the action link "View Attachments". If this link does not appear, there are no attachment files.]

To ensure your figures meet our technical requirements, please review our figure guidelines: https://journals.plos.org/plosone/s/figures

You may also use PLOS’s free figure tool, NAAS, to help you prepare publication quality figures: https://journals.plos.org/plosone/s/figures#loc-tools-for-figure-preparation.

NAAS will assess whether your figures meet our technical requirements by comparing each figure against our figure specifications.

Attachment Submitted filename: PONE-D-26-12466 (1) Jyoti.pdf

# Author comment: Author response to Decision Letter 1

**DOI:** 10.1371/journal.pone.0353975.r002

24 Apr 2026

Editor in Chief

Thank you very much for the reviews on our manuscript, entitled “Comparative Evaluation of Root Canal Obturation Techniques and Sealers in Diabetic Dentin: An in-vitro Push-Out Bond Strength Test’’ by myself, Asst. Prof. Mevlüt Sinan OCAK, Fırat University, Faculty of Dentistry, Department of Endodontics, Elazığ, Turkey.

We thank the reviewers for their helpful comments. We have made substantial changes in the manuscript based on the reviewers’ comments. Our responses and revisions made are shown in the attached file. The associated amendments are written in highlighted in the text to make them stand out. I have added this information to the acknowledgements section. After adding the corrections and requested references to the article, I have revised all the references I used and updated their order. We believe that the manuscript is now suitable for publication in Journal of Stomatology oral and Maxillofacial Surgery.

Sincerely yours,

Reviewers' Comments to Author:

Reviewer 1

We are grateful for your unbiased and constructive criticism. All changes made to the manuscript marked or yellow highlighted in the revised manuscript. Some references added due to referee’s suggested statements. Best regards.

No Reviewer’s comments Author responses

1. Kindly do a english proof reading We thank the reviewer for this helpful comment. A final comprehensive proofreading of the manuscript has been performed to correct minor grammatical inconsistencies and improve overall language quality.

2. Add recent article in citation We thank the reviewer for this valuable suggestion. Recent and relevant studies published in the last five years have been.

3. Mention the limitation before the conclusion is mentioned was age of patients taken into consideration? We thank the reviewer for this comment. The limitations sentence has been revised to clarify that age and gender were not included as controlled variables within the scope of the study design.

“First, the specimens were collected from diabetic patients of variable ages and disease durations, and age and gender were not included as controlled variables within the scope of this experimental design, which may have contributed to heterogeneity in dentin structure and mechanical behavior.9,13 The influence of age and gender on the results was not evaluated.”

4. Was the gender noted when the teeth were selected for extraction? would age and gender of the patient influence the results obtained ? was it checked? We thank the reviewer for this important comment. Age and gender were not included as variables in this study, and their potential influence on the results was not evaluated. This has now been clearly acknowledged in the limitations section.

Reviewer 2

We are grateful for your unbiased and constructive criticism. All changes made to the manuscript marked or turquoise highlighted in the revised manuscript.

No Reviewer’s comments Author responses

1. The groups have different techniques and different materials (sealers) used. This adds to the confounding factor to the statistical analysis. It is like comparing oranges with apples.

If for first and second group, the bioceramic sealer was used, then it would be unbiased comparison between the obturation techniques, according to the aim of the study. We thank the reviewer for this insightful comment. We agree that using different obturation techniques together with different sealer materials introduces multiple variables that may influence the outcomes. However, the primary aim of the present study was not to isolate the effect of obturation technique alone, but rather to evaluate clinically relevant obturation approaches as they are commonly applied in practice, where specific techniques are typically used in combination with particular types of sealers.

In this context, the combinations tested in the present study reflect real-world clinical scenarios rather than isolated variables. Additionally, a sealer-only group was included to partially isolate and evaluate the intrinsic bonding ability of the bioceramic material, thereby providing further insight into the role of the sealer independent of gutta-percha.

We acknowledge that a fully standardized comparison using the same sealer across all techniques could provide additional information and should be considered in future studies. This limitation has now been clarified in the manuscript.

“Another limitation is that obturation techniques were evaluated in combination with different sealer materials, which does not allow complete isolation of the individual effect of each variable. However, this approach was intentionally adopted to reflect clinically relevant treatment strategies, where specific techniques are commonly used with particular types of sealers.”

Response to Editor:

We confirm that the minimal dataset required to reproduce the results of this study is available. The underlying raw data, including the individual values used to calculate means and standard deviations, have been provided as a Supporting Information file (S1 Data). The Data Availability Statement has been updated accordingly.

Sincerely yours,

Response to Editor:

Dear Editor,

Thank you for your message. We confirm that the Supporting Information file “S1 Data.xlsx” contains the complete minimal dataset required to reproduce the results of our study.

This dataset includes all individual raw measurements used to calculate the reported means, standard deviations, and statistical analyses. Specifically, it contains the push-out bond strength values (MPa) for each specimen, including group allocation and root region (coronal, middle, apical).

No additional data were used beyond those provided in this file to generate the results presented in the manuscript.

We confirm that the dataset is sufficient to fully reproduce the findings of the study.

Kind regards,

Dr. Mevlüt Sinan Ocak

Attachment Submitted filename: Response to Reviewer - 2.docx

# Peer review: Decision Letter 1

**DOI:** 10.1371/journal.pone.0353975.r003

**Authors:** Mohmed Isaqali Karobari, Mohmed Isaqali Karobari

1 Jul 2026

Comparative Evaluation of Root Canal Obturation Techniques and Sealers in Diabetic Dentin: An in-vitro Push-Out Bond Strength Test

PONE-D-26-12466R1

Dear Dr. OCAK,

We’re pleased to inform you that your manuscript has been judged scientifically suitable for publication and will be formally accepted for publication once it meets all outstanding technical requirements.

Within one week, you’ll receive an e-mail detailing the required amendments. When these have been addressed, you’ll receive a formal acceptance letter and your manuscript will be scheduled for publication.

An invoice will be generated when your article is formally accepted. Please note, if your institution has a publishing partnership with PLOS and your article meets the relevant criteria, all or part of your publication costs will be covered. Please make sure your user information is up-to-date by logging into Editorial Manager at Editorial Manager® and clicking the ‘Update My Information' link at the top of the page. For questions related to billing, please contact billing support.

If your institution or institutions have a press office, please notify them about your upcoming paper to help maximize its impact. If they’ll be preparing press materials, please inform our press team as soon as possible -- no later than 48 hours after receiving the formal acceptance. Your manuscript will remain under strict press embargo until 2 pm Eastern Time on the date of publication. For more information, please contact onepress@plos.org.

Kind regards,

Mohmed Isaqali Karobari, BDS, MScD.Endo, Ph.D. Endo, FDS, FPFA, FICD, MFDS

Academic Editor

PLOS One

Additional Editor Comments (optional):

Dear Authors,

The authors have addressed all the reviewers' comments and suggestions, and the manuscript has undergone significant improvement. I would like to congratulate the authors and wish them all the very best in their future endeavours.

Best regards and keep well

Reviewers' comments:

Reviewer's Responses to Questions

**Comments to the Author**

1. If the authors have adequately addressed your comments raised in a previous round of review and you feel that this manuscript is now acceptable for publication, you may indicate that here to bypass the “Comments to the Author” section, enter your conflict of interest statement in the “Confidential to Editor” section, and submit your "Accept" recommendation.

Reviewer #2: All comments have been addressed

**********

2. Is the manuscript technically sound, and do the data support the conclusions?

The manuscript must describe a technically sound piece of scientific research with data that supports the conclusions. Experiments must have been conducted rigorously, with appropriate controls, replication, and sample sizes. The conclusions must be drawn appropriately based on the data presented.

Reviewer #2: Yes

**********

3. Has the statistical analysis been performed appropriately and rigorously?

Reviewer #2: Yes

**********

4. Have the authors made all data underlying the findings in their manuscript fully available?

The PLOS Data policy requires authors to make all data underlying the findings described in their manuscript fully available without restriction, with rare exception (please refer to the Data Availability Statement in the manuscript PDF file). The data should be provided as part of the manuscript or its supporting information, or deposited to a public repository. For example, in addition to summary statistics, the data points behind means, medians and variance measures should be available. If there are restrictions on publicly sharing data—e.g. participant privacy or use of data from a third party—those must be specified.

Reviewer #2: Yes

**********

5. Is the manuscript presented in an intelligible fashion and written in standard English?

PLOS ONE does not copyedit accepted manuscripts, so the language in submitted articles must be clear, correct, and unambiguous. Any typographical or grammatical errors should be corrected at revision, so please note any specific errors here.

Reviewer #2: Yes

**********

6. Review Comments to the Author

Please use the space provided to explain your answers to the questions above. You may also include additional comments for the author, including concerns about dual publication, research ethics, or publication ethics. (Please upload your review as an attachment if it exceeds 20,000 characters)

Reviewer #2: The authors have addressed my concerns regarding the study groups and I am satisfied with their explanation. The study provides valuable insights as far as the selection of root canal sealer is concerned. If the authors would have designed their major groups of obturation techniques and then made subgroups in each technique for two experimental sealers, the results would have been more contextual and conclusive. However, overall, the manuscript is well structured, insightful and study design/conclusions are relevant to routine contemporary endodontic clinical practice.

**********

7. PLOS authors have the option to publish the peer review history of their article (what does this mean?). If published, this will include your full peer review and any attached files.

If you choose “no”, your identity will remain anonymous but your review may still be made public.

**Do you want your identity to be public for this peer review?**  For information about this choice, including consent withdrawal, please see our Privacy Policy.

Reviewer #2: **Yes:** Dr. Jyoti Mandlik

**********

# Editor report: Acceptance letter

**DOI:** 10.1371/journal.pone.0353975.r004

**Authors:** Mohmed Isaqali Karobari, Mohmed Isaqali Karobari

PONE-D-26-12466R1

PLOS One

Dear Dr. OCAK,

I'm pleased to inform you that your manuscript has been deemed suitable for publication in PLOS One. Congratulations! Your manuscript is now being handed over to our production team.

At this stage, our production department will prepare your paper for publication. This includes ensuring the following:

* All references, tables, and figures are properly cited

* All relevant supporting information is included in the manuscript submission,

* There are no issues that prevent the paper from being properly typeset

You will receive further instructions from the production team, including instructions on how to review your proof when it is ready. Please keep in mind that we are working through a large volume of accepted articles, so please give us a few days to review your paper and let you know the next and final steps.

Lastly, if your institution or institutions have a press office, please let them know about your upcoming paper now to help maximize its impact. If they'll be preparing press materials, please inform our press team within the next 48 hours. Your manuscript will remain under strict press embargo until 2 pm Eastern Time on the date of publication. For more information, please contact onepress@plos.org.

You will receive an invoice from PLOS for your publication fee after your manuscript has reached the completed accept phase. If you receive an email requesting payment before acceptance or for any other service, this may be a phishing scheme. Learn how to identify phishing emails and protect your accounts at https://explore.plos.org/phishing.

If we can help with anything else, please email us at customercare@plos.org.

Thank you for submitting your work to PLOS ONE and supporting open access.

Kind regards,

PLOS ONE Editorial Office Staff

on behalf of

Prof Dr. Mohmed Isaqali Karobari

Academic Editor

PLOS One
